# Single-drug versus combination antimicrobial therapy in critically ill patients with hospital-acquired pneumonia and ventilator-associated pneumonia due to Gram-negative pathogens: a multicenter retrospective cohort study

**DOI:** 10.1186/s13054-023-04792-0

**Published:** 2024-01-03

**Authors:** François Barbier, Claire Dupuis, Niccolò Buetti, Carole Schwebel, Élie Azoulay, Laurent Argaud, Yves Cohen, Vivien Hong Tuan Ha, Marc Gainnier, Shidasp Siami, Jean-Marie Forel, Christophe Adrie, Étienne de Montmollin, Jean Reignier, Stéphane Ruckly, Jean-Ralph Zahar, Jean-François Timsit, the OutcomeRéa Study Group

**Affiliations:** 1 Médecine Intensive Réanimation, Centre Hospitalier Universitaire d’Orléans, Orléans, France; 2 Médecine Intensive Réanimation, Centre Hospitalier Universitaire Gabriel Montpied, Clermont-Ferrand, France; 3 Infection Control Programme, University of Geneva Hospitals and Faculty of Medicine, Geneva, Switzerland; 4 IAME UMR 1137, INSERM, Université Paris-Cité, Paris, France; 5 Médecine Intensive Réanimation, Centre Hospitalier Universitaire Grenoble – Alpes, La Tronche, France; 6 Médecine Intensive Réanimation, Centre Hospitalier Universitaire Saint-Louis, Assistance Publique – Hôpitaux de Paris, Paris, France; 7 Médecine Intensive Réanimation, Centre Hospitalier Universitaire Edouard Herriot, Hospices Civils de Lyon, Lyon, France; 8 Médecine Intensive Réanimation, Centre Hospitalier Universitaire Avicenne, Assistance Publique – Hôpitaux de Paris, Bobigny, France; 9 Réanimation Médicale, Grand Hôpital de L’Est Parisien, Meaux, France; 10 Réanimation des Urgences, Centre Hospitalier Universitaire La Timone, Assistance Publique – Hôpitaux de Marseille, Marseille, France; 11 Réanimation Polyvalente, Centre Hospitalier Sud-Essonne, Étampes, France; 12 Médecine Intensive Réanimation, Centre Hospitalier Universitaire Nord, Assistance Publique – Hôpitaux de Marseille, Marseille, France; 13 Réanimation Polyvalente, Centre Hospitalier Delafontaine, Saint-Denis, France; 14 Service de Médecine Intensive et Réanimation Infectieuse, Centre Hospitalier Universitaire Bichat – Claude Bernard, Assistance Publique – Hôpitaux de Paris, Paris, France; 15 Médecine Intensive Réanimation, Centre Hospitalier Universitaire de Nantes, Nantes, France; 16 Département de Biostatistiques, OutcomeRéa, Paris, France; 17 Département de Microbiologie Clinique, Centre Hospitalier Universitaire Avicenne, Assistance Publique – Hôpitaux de Paris, Bobigny, France; 18 Service de Médecine Intensive Réanimation, Centre Hospitalier Universitaire d’Orléans, 14, Avenue de L’Hôpital, 45000 Orléans, France

**Keywords:** Antimicrobial therapy, Ventilator-associated pneumonia, Hospital-acquired pneumonia, Enterobacterales, *Pseudomonas aeruginosa*, Intensive care unit, Antimicrobial stewardship, De-escalation, Outcome

## Abstract

**Key messages:**

In this study including 391 critically ill patients with nosocomial pneumonia due to Gram-negative pathogens, combination therapy was not associated with a reduced hazard of death at Day 28 or a greater likelihood of clinical cure at Day 14. No over-risk of AKI was observed in patients receiving combination therapy.

**Background:**

The benefits and harms of combination antimicrobial therapy remain controversial in critically ill patients with hospital-acquired pneumonia (HAP), ventilated HAP (vHAP) or ventilator-associated pneumonia (VAP) involving Gram-negative bacteria.

**Methods:**

We included all patients in the prospective multicenter OutcomeRea database with a first HAP, vHAP or VAP due to a single Gram-negative bacterium and treated with initial adequate single-drug or combination therapy. The primary endpoint was Day-28 all-cause mortality. Secondary endpoints were clinical cure rate at Day 14 and a composite outcome of death or treatment-emergent acute kidney injury (AKI) at Day 7. The average effects of combination therapy on the study endpoints were investigated through inverse probability of treatment-weighted regression and multivariable regression models. Subgroups analyses were performed according to the resistance phenotype of the causative pathogens (multidrug-resistant or not), the pivotal (carbapenems or others) and companion (aminoglycosides/polymyxins or others) drug classes, the duration of combination therapy (< 3 or ≥ 3 days), the SOFA score value at pneumonia onset (< 7 or ≥ 7 points), and in patients with pneumonia due to non-fermenting Gram-negative bacteria, pneumonia-related bloodstream infection, or septic shock.

**Results:**

Among the 391 included patients, 151 (38.6%) received single-drug therapy and 240 (61.4%) received combination therapy. VAP (overall, 67.3%), vHAP (16.4%) and HAP (16.4%) were equally distributed in the two groups. All-cause mortality rates at Day 28 (overall, 31.2%), clinical cure rate at Day 14 (43.7%) and the rate of death or AKI at Day 7 (41.2%) did not significantly differ between the groups. In inverse probability of treatment-weighted analyses, combination therapy was not independently associated with the likelihood of all-cause death at Day 28 (adjusted odd ratio [aOR], 1.14; 95% confidence interval [CI] 0.73–1.77; *P* = 0.56), clinical cure at Day 14 (aOR, 0.79; 95% CI 0.53–1.20; *P* = 0.27) or death or AKI at Day 7 (aOR, 1.07; 95% CI 0.71–1.63; *P* = 0.73). Multivariable regression models and subgroup analyses provided similar results.

**Conclusions:**

Initial combination therapy exerts no independent impact on Day-28 mortality, clinical cure rate at Day 14, and the hazard of death or AKI at Day 7 in critically ill patients with mono-bacterial HAP, vHAP or VAP due to Gram-negative bacteria.

**Supplementary Information:**

The online version contains supplementary material available at 10.1186/s13054-023-04792-0.

## Introduction

The potential benefits and harms of initial combination antimicrobial therapy remain controversial in patients with hospital-acquired pneumonia (HAP) and ventilator-associated pneumonia (VAP) [1–5]. Beyond the enhanced probability of administering at least one adequate agent in patients at-risk for multidrug-resistant bacteria (MDRB), combination therapy (usually a β-lactam plus an aminoglycoside or a fluoroquinolone) could conceptually improve bacterial clearance when both drugs are active against the causative pathogen, thereby hastening infection resolution and preventing the emergence of resistant mutants [6]. Nevertheless, most of randomized trials and observational studies addressing this issue reported no improvement in survival, treatment success rate or others patient-centered outcomes with combination therapy [7–9], including in patients with pneumonia due to potentially difficult-to-treat pathogens such as *Pseudomonas aeruginosa* [10–12]. In addition, combination therapy has been linked with an increased hazard of adverse events, especially acute kidney injury (AKI) when aminoglycosides are used as companion drugs [13, 14].

Yet, the overall quality of evidence on this question is low due to heterogeneity in study populations, pathogen distribution, and antimicrobial regimen [3, 7]. Further, most of dedicated studies enrolled a low proportion of patients with septic shock or severe comorbid conditions. To date, initial combination therapy is still recommended in the most severely ill patients with HAP or VAP, notably those infected with *P. aeruginosa* [4, 5]. Certain works suggest that the prognostic impact of combination therapy could depend on initial severity, with a survival benefit compared to single-drug therapy in patients with the highest baseline risk of death [15–17] and, conversely, a deleterious effect in those with a low probability of dying at infection onset [15].

The primary objective of this retrospective study based on prospectively collected data was to investigate the effect of initial adequate combination therapy compared to adequate single-drug therapy on Day-28 all-cause mortality in critically ill patients with HAP, ventilated HAP (vHAP) or VAP due to Gram-negative bacteria. The secondary objectives were to appraise the impact of combination therapy on clinical cure rates at Day 14 and the hazard of death or AKI at Day 7.

## Patients and methods

### Study design and data source

This observational study was conducted using the OutcomeRéa prospective database fueled since 1996 by a total of 32 intensive care units (ICU) in France, including 18 ICUs located in university hospitals. The methodology implemented for data collection and quality control has been extensively described elsewhere [18]. The database protocol was submitted to the Institutional Review Board of the Clermont-Ferrand University Hospital (Clermont-Ferrand, France) who waived the need for informed consent (IRB no. 5891). The OutcomeRéa database has been approved by the French Advisory Committee for Data Processing in Health Research and registered by the French National Informatics and Liberty Commission (registration n°8999262), in compliance with French law on electronic data sources. The methods and results of this study are exposed according to the STROBE guidelines [19].

### Study population and definitions

All patients with a first ICU stay between January 1st, 2008 and September 1st, 2019 were considered for enrollment in the study. Among them, we included those with a monobacterial HAP, vHAP or VAP due to Gram-negative bacteria and treated with adequate single-drug or adequate combination antimicrobial therapy at Day 0 (date of pneumonia diagnosis) and/or Day 1. Only the first pneumonia was analyzed in patients with multiple episodes during the ICU stay. Pneumonia cases involving more than one Gram-negative pathogen were not retained as each isolate may exhibit a distinct antimicrobial susceptibility phenotype, thereby confusing the categorization into adequate single-drug therapy and adequate combination therapy (see below). Given the study objectives exposed above, patients not receiving initial adequate therapy were not considered.

HAP was defined as pneumonia occurring more than 48 h after hospital admission in patients not receiving invasive mechanical ventilation (MV): this category included both ICU-acquired non-ventilator-associated pneumonia and non-ICU-acquired HAP requiring ICU admission. HAP requiring tracheal intubation and MV between Day -1 and Day 2 were defined as ventilated HAP (vHAP) [20]—hereafter, the term HAP will only refer to HAP not requiring MV. VAP were defined as pneumonia occurring in patients receiving MV for more than 48 h. HAP, vHAP and VAP episodes were prospectively entered in the database by the attending ICU physicians provided that standardized diagnostic criteria were met, that is (i) new or persistent/progressive pulmonary infiltrates on chest X-ray and/or CT scan, combined with (ii) purulent sputum or tracheal aspirates, (iii) fever or hypothermia (body temperature ≥ 38.5 °C or ≤ 36.5 °C, respectively) and/or leukocytosis or leukopenia (white blood cells count ≥ 10^4^ mL or ≤ 4.10^3^ mL, respectively), and (iv) a positive quantitative lower respiratory tract sample (endotracheal aspirate ≥ 10^5^ colony-forming unit [CFU]/mL, broncho-alveolar lavage fluid ≥ 10^4^ CFU/mL, or plugged telescopic catheter ≥ 10^3^ CFU/mL). Only pneumonia due to a single Gram-negative pathogen were analyzed.

Adequate antimicrobial therapy was defined as the administration of one (single-drug group) or two (combination group) agents with in vitro activity against the causative pathogen, whatever the antimicrobial classes. For combination regimen, the companion drug was defined as the first discontinued antimicrobial (de-escalation) while the drug class pursued as definite therapy was defined as pivotal [21]. In all participating centers, the reinjection of aminoglycosides, when deemed indicated, was performed according to trough concentration monitoring.

MDR, extensively drug-resistant (XDR) and pan-drug-resistant (MDR) bacteria were defined according to the Centers for Disease Control and Prevention and the European Center for Disease Control and Prevention classification [22]. Immune deficiency was defined as any form of immunosuppression excepting HIV infection without acquired immune deficiency syndrome (AIDS) (that is, AIDS, active or recent < 5 years solid or hematological neoplasms, solid organ or bone marrow transplantation, and current or recent administration of corticosteroids [more than 0,5 mg/kg/day of equivalent prednisolone for more than 3 months] and/or other immunosuppressive drugs). Sepsis and septic shock were defined according to the Sepsis-3 criteria [23]. Pneumonia-related bloodstream infection (BSI) was defined as one or more blood cultures collected between Day -2 and Day 2 and growing the same pathogen than the one responsible for pneumonia.

The primary study endpoint was all-cause mortality at Day 28 [24]. Secondary endpoints were clinical cure rate at Day 14 and a composite outcome of death or treatment-emergent AKI at Day 7.

Clinical cure was defined as the complete resolution of all initial clinical and biological signs of pneumonia at Day 14, that is normal body temperature, white blood cell count between 4.10^9^ and 12.10^9^/L, increase in PaO_2_/FiO_2_ ratio ≥ 50 mmHg under MV or successful extubation, vasopressor weaning (when administered at Day 0 and/or Day 1) and, for patients with HAP, respiratory rate < 25/min if > 25/min at Day 0 and/or Day 1. Death from any cause at Day 14, bloodstream infection due the pathogen responsible for pneumonia between Day 7 and Day 14, and a lower respiratory tract sample positive with this pathogen above the significance threshold between Day 7 and Day 14 were classified as clinical failure. Patients discharged alive from the ICU before Day 14 were considered as clinically cured. AKI was defined according to the KDIGO criteria, with or without new requirement for renal replacement therapy [25]—episodes occurring between Day 1 and Day 7 were defined as treatment-emergent AKI. As early death may act as a competing event for the development of AKI at Day 7, both outcomes were analyzed as a composite endpoint, similarly to recent studies focused on major adverse kidney events [26].

### Statistical analyses

Data are expressed as number (percentage) for categorical variables and median (interquartile range) for continuous variables, unless otherwise indicated. Categorical and continuous variables were compared between the single-drug and combination groups using the Fisher’s exact test or the χ^2^ test and the Kruskal–Wallis test or the T-test, respectively.

The adjusted odd ratios (aOR) and their 95% confidence intervals (CI) for the association between antimicrobial regimen (i.e., single drug versus combination) and the study endpoints (all-cause mortality at Day 28, clinical cure rate at Day 14, and death or treatment-emergent AKI at Day 7) were estimated using logistic regressions. Two separate approaches were used to estimate the average treatment effect of combination therapy while accounting for confounding: (i) inverse probability of treatment-weighted (IPTW) regression; and (ii) multivariable regression. Propensity scores (PS) were calculated from selected prognostically important covariates (those related to outcomes) and confounding covariates (those related to both antimicrobial regimen and outcomes) through multivariable logistic regressions (Table S1 in the Additional file 1). The concordance statistic (c-statistic) was used to test the appropriateness of the models. The IPTW were defined as the inverse of the PS for patients receiving combination therapy and 1/(1–PS) (i.e., the inverse of 1–PS) those receiving single-drug therapy. Stabilized weights (defined as the weight multiplied by the probability of receiving the treatment actually administered) were calculated from the PS [27]. After calculating the weights, absolute standardized differences were measured for each covariate to evaluate the success of the models in creating balanced cohorts (Figure S1 in the Additional file 1). Standardized differences of more than 0.1 were considered an indicative of imbalance. IPTW-adjusted Day-28 survival curves were built and compared between the two groups, with calculation of the weighted hazard ratio and its 95% CI.

The same covariates than those selected for the IPTW models were used for the multivariable regression models. Subgroup analyses based on these multivariable regression models were then performed according to the resistance phenotype of the causative pathogens (MDR or non-MDR), the pivotal β-lactam class (carbapenems or others), the companion drug class (aminoglycosides or others), the duration of combination therapy (< 3 or ≥ 3 days), the SOFA score value at pneumonia onset (< 7 and ≥ 7 points), in patients infected with non-fermenting Gram-negative bacteria, in those with pneumonia-related BSI, and in those presenting with septic shock at pneumonia onset.

Statistical analyses were performed using SAS 9.4 software© (SAS Institute, Cary, NC, USA). A *P*-value less than 0.05 was considered significant.

## Results

### Study population

A total of 488 patients with HAP or VAP due to Gram-negative bacteria and receiving initial adequate antimicrobial therapy were identified over the inclusion period, including 97 patients who were excluded owing to polymicrobial pneumonia (Figure S2 in the Additional file 1). The remaining 391 patients with mono-bacterial pneumonia were included in the study; among them, 151 (38.6%) received initial adequate single-drug therapy and 240 (61.4%) received initial adequate combination therapy. The proportion of patients treated with combination therapy decreased over the inclusion period (Table 1). The two groups did not significantly differ in terms of prevalence of chronic conditions (especially immune deficiency [overall, 23.8%] and renal comorbidities [6.4%]), SAPS 2 values at ICU admission (57 [37–64]), initial requirement for organ support (invasive mechanical ventilation, 83.4%; vasopressors, 58.3%; renal replacement therapy, 17.4%), and rate of MDRB carriage prior to pneumonia onset (11.0%) (Table 1).

**Table 1:** Main characteristics of the study population

	All patients with pneumonia (n = 391)	Patients treated with adequate single-drug therapy (n = 151)	Patients treated with adequate combination therapy (n = 240)	*P*-value
**Admission period**				
2008–2011 2012–2015 2016–2019	174 (44.5) 161 (41.2) 56 (14.3)	58 (38.4) 58 (38.4) 35 (23.2)	116 (48.3) 103 (42.9) 21 (8.8)	0.0006
**Male sex**	281 (71.9)	103 (68.2)	178 (74.2)	0.20
**Age, years**	65 (54–73)	63 (53–73)	66 (55–73)	0.89
BMI, kg.m^−2^	24.9 (21.5–29.7)	25.1 (22.5–30)	24.8 (21.4–29.1)	0.51
**Chronic diseases**				
Any, except immune deficiency Hepatic Cardiac Respiratory Renal Immune deficiency	163 (41.7) 23 (5.9) 78 (19.9) 75 (19.2) 25 (6.4) 93 (23.8)	60 (39.7) 7 (4.6) 29 (19.2) 31 (20.5) 8 (5.3) 35 (23.2)	103 (42.9) 16 (6.7) 49 (20.4) 44 (18.3) 17 (7.1) 58 (24.2)	0.53 0.41 0.77 0.59 0.48 0.82
**SAPS 2 at ICU admission**	50 (37–64)	53 (38–64)	48 (35–64)	0.35
**SOFA score at ICU admission**	8 (5–11)	8 (5–11)	8 (5–11)	0.42
**Organ support at ICU admission** ^a^				
Non-invasive ventilation and/or HFNO Invasive MV Vasopressors ECMO RRT	28 (7.2) 326 (83.4) 228 (58.3) 16 (4.1) 68 (17.4)	10 (6.6) 126 (83.4) 81 (53.6) 7 (4.6) 25 (16.6)	18 (7.5) 200 (83.3) 147 (61.3) 9 (3.8) 43 (17.9)	0.84 1 0.14 0.79 0.78
**Sepsis at ICU admission**	248 (63.4)	83 (55.0)	165 (68.8)	0.006
**Septic shock at ICU admission**	117 (29.9)	37 (24.5)	80 (33.3)	0.06
**Antimicrobial exposure before pneumonia** ^b^				
Non-antipseudomonal β-lactams Antipseudomonal β-lactams, except carbapenems Antipseudomonal carbapenems Fluoroquinolones Aminoglycosides Glycopeptides	102 (26.1) 73 (18.7) 25 (6.4) 23 (5.9) 123 (31.5) 56 (14.3)	42 (27.8) 26 (17.2) 9 (6.0) 7 (4.6) 44 (29.1) 23 (15.2)	60 (25.0) 47 (19.6) 16 (6.7) 16 (6.7) 79 (32.9) 33 (13.8)	0.54 0.56 0.78 0.41 0.43 0.68
**MDRB carriage before pneumonia**	43 (11.0)	12 (7.9)	31 (12.9)	0.13
**Pneumonia classification**				
VAP vHAP HAP	263 (67.3) 64 (16.4) 64 (16.4)	105 (69.5) 18 (11.9) 28 (18.6)	158 (65.8) 46 (19.2) 36 (15.0)	0.15
**Pneumonia characteristics**				
Time from hospital admission Time from hospital admission > 7 days Time from ICU admission PaO_2_/FiO_2_ < 200 mmHg ^c^ Sepsis ^c^ Septic shock ^c^ SOFA score value ^c^ ΔSOFA from Day-2 to Day 2 Pneumonia-related BSI	12 (7–22) 275 (70.3) 8 (4–15) 196 (50.1) 312 (79.8) 99 (25.3) 7 (4–9) 2 (1–5) 28 (7.2)	10 (6–20) 97 (64.2) 8 (3–13) 72 (47.7) 112 (74.2) 30 (19.9) 6 (4–9) 2 (1–4) 10 (6.6)	14 (7–23) 178 (74.2) 8 (4–15.5) 124 (51.7) 200 (83.3) 69 (28.8) 7 (4–9) 2 (1–5) 18 (7.5)	0.02 0.04 0.41 0.44 0.03 0.05 0.16 0.04 0.74
**Gram-negative bacteria responsible for pneumonia**				
Enterobacterales *Klebsiella pneumoniae*/*Klebsiella oxytoca* * Escherichia coli* * Serratia marcescens* * Klebsiella aerogenes* * Enterobacter cloacae* Others Non-fermenters * Pseudomonas aeruginosa* * Stenotrophomonas maltophilia* *Achromobacter* spp *Acinetobacter baumannii* *Haemophilus* spp MDR bacterium XDR/PDR bacterium	207 (52.9) 60 (15.4) 57 (14.6) 45 (11.5) 13 (3.3) 9 (2.3) 23 (5.9) 170 (43.5) 150 (38.4) 10 (2.6) 6 (1.5) 4 (1.0) 14 (3.6) 76 (19.5) 8 (2.1)	91 (60.3) 24 (15.8) 22 (14.6) 28 (18.5) 6 (4.0) 2 (1.3) 9 (6.1) 52 (34.4) 44 (29.2) 3 (2.0) 4 (2.6) 1 (0.7) 8 (5.3) 24 (15.9) 3 (2.0)	116 (48.3) 36 (15.1) 35 (14.6) 17 (7.1) 7 (2.9) 7 (2.9) 14 (5.9) 118 (49.2) 106 (44.2) 7 (2.9) 2 (0.8) 3 (1.3) 6 (2.5) 52 (21.7) 5 (2.1)	0.02 0.89 1 0.0009 0.57 0.49 1 0.005 0.003 0.75 0.21 1 0.17 0.19 1
**Duration of antimicrobial therapy, overall, days**	8 (6–12)	7 (5–10)	8.5 (6–13)	0.04
**Duration of combination therapy, days**	3 (2–5)	-	3 (2–5)	-
**Pivotal antimicrobial agent** Antipseudomonal penicillins ± BLI Antipseudomonal carbapenems Antipseudomonal cephalosporins Non-antipseudomonal penicillins/cephalosporins Aztreonam Fluoroquinolones Aminoglycosides Cotrimoxazole Colistin	137 (35.0) 104 (26.6) 76 (19.5) 55 (14.1) 1 (0.3) 9 (2.3) 3 (0.8) 3 (0.8) 3 (0.8)	47 (31.1) 32 (21.2) 25 (16.6) 37 (24.5) 0 3 (2.0) 3 (2.0) ^d^ 3 (2.0) 1 (0.7) ^e^	90 (37.5) 72 (30.0) 51 (21.2) 18 (7.5) 1 (0.4) 6 (2.5) 0 0 2 (0.8)	< 0.0001
**Companion antimicrobial agent** Aminoglycosides Fluoroquinolones Cotrimoxazole Colistin	174 (72.5) 51 (21.3) 12 (5) 3 (1.3)	- - - -	174 (72.5) 51 (21.3) 12 (5) 3 (1.3)	-
**Post-pneumonia follow-up, days**	31 (13–135.5)	34 (12–136)	31 (13–135)	0.87
**Pneumonia outcomes** Clinical cure at Day 14 Relapse Time to relapse, days Superinfection Time to superinfection, days	171 (43.7) 37 (9.5) 15 (10–18) 10 (2.6) 15.5 (9–30)	75 (49.7) 8 (5.3) 15 (7.5–18) 3 (2.0) 14 (2–15)	96 (40.0) 29 (12.1) 13 (10–18) 7 (2.9) 29 (9–37)	0.06 0.03 0.50 0.57 0.19
**Treatment-emergent AKI at Day 7** Need for RRT	142 (36.3) 43 (30.3)	47 (31.1) 10 (21.3)	95 (39.6) ^f^ 33 (34.7)	0.09 0.10
**MDRB carriage acquired after pneumonia** ^b^	57 (14.6)	29 (19.2)	28 (11.7)	0.04
*Clostridioides difficile* infection after pneumonia ^b^	5 (1.3)	4 (2.6)	1 (0.4)	0.10
**Organ support during the ICU stay** MV Duration, days Vasopressors Duration, days RRT Duration, days ECMO Duration, days	376 (96.2) 18 (11–29) 308 (78.8) 7 (4–13) 144 (36.8) 8.5 (3–16) 25 (6.4) 12 (5–15)	141 (93.4) 17 (10–27) 106 (70.2) 7 (4–13) 56 (37.1) 8.5 (2.5–15.5) 9 (6.0) 11 (9–12)	235 (97.9) 19 (12–30) 202 (84.2) 7 (4–14) 88 (36.7) 8.5 (4–16) 16 (6.7) 13.5 (4.5–18.5)	0.03 0.78 0.001 0.89 0.93 0.86 0.78 0.68
**ICU LOS, days**	23 (14–39)	22 (13–37)	26 (15–41)	0.55
**Hospital LOS, days**	40 (24–69)	40.5 (22.5–70)	40 (26–69)	0.66
**In-hospital death**				
Day 7 Day 14 Day 28 Overall	40 (10.2) 86 (22.0) 122 (31.2) 195 (49.9)	16 (10.6) 34 (22.5) 43 (28.5) 64 (42.4)	24 (10.0) 52 (21.7) 79 (32.9) 131 (54.6)	0.85 0.84 0.36 0.02

Data are exposed as number (percentage) or median (interquartile range)

BMI, body mass index; SAPS 2, simplified acute physiology score 2; ICU, intensive care unit; SOFA, sepsis-related organ failure assessment; HFNO, high-flow nasal oxygen; MV, mechanical ventilation; ECMO, extra-corporeal membrane oxygenation; RRT, renal replacement therapy; BSI, bloodstream infection; MDRB, multidrug-resistant bacteria; VAP, ventilator-associated pneumonia; vHAP, ventilated hospital-acquired pneumonia; HAP, non-ventilated hospital-acquired pneumonia; XDR, extensively drug-resistant; PDR, pandrug-resistant; BLI, β-lactamase inhibitor

^a^First 48 h of the ICU stay

^b^During the ICU stay

^c^At pneumonia onset, *i.e.* Day 0 (date of pneumonia diagnosis) and/or Day 1

^d^Pneumonia due to MDR *P. aeruginosa* (n = 2) and MDR *E. coli* (n = 1)

^e^Pneumonia due to MDR *P. aeruginosa*

^f^66/174 (37.9%) for aminoglycoside-containing combinations versus 29/66 (43.9%) for other combinations (*P* = 0.30)

### Characteristics of pneumonia and antimicrobial therapy

The two groups were similar regarding pneumonia distribution (overall, VAP, vHAP and HAP, 67.3%, 16.4% and 16.4% of episodes, respectively), SOFA score values at pneumonia onset (7 [4–9] points), proportion of cases with a PaO_2_/FiO_2_ ratio < 200 mmHg (50.1%), and prevalence of MDR (19.5%) and XDR/PDR (2.1%) pathogens (Table 1). Pneumonia due to *P. aeruginosa* or other non-fermenting Gram-negative bacteria (49.2% versus 34.4%, *P* = 0.005) and pneumonia-related sepsis (83.3% versus 74.2%, *P* = 0.03) were more common in the combination group. Pneumonia-related BSI was documented in 28 patients (7.2%).

β-lactams were prescribed as pivotal drugs in 372 patients (95.1%) (Table 1). Antipseudomonal β-lactams—especially carbapenems—were more frequently used in the combination group (*P* < 0.0001). Companion drugs, mostly aminoglycosides (72.5%) and fluoroquinolones (21.3%), were administered for a median duration of 3 (2–5) days. The total duration of antimicrobial therapy was longer in the combination group than in the single-drug group (8.5 [6–13] versus 7 [5–10] days, *P* = 0.04). Initial dosing schemes are exposed in Additional file 1: Table S2.

### Impact of combination therapy on day-28 mortality

**Fig. 1:**
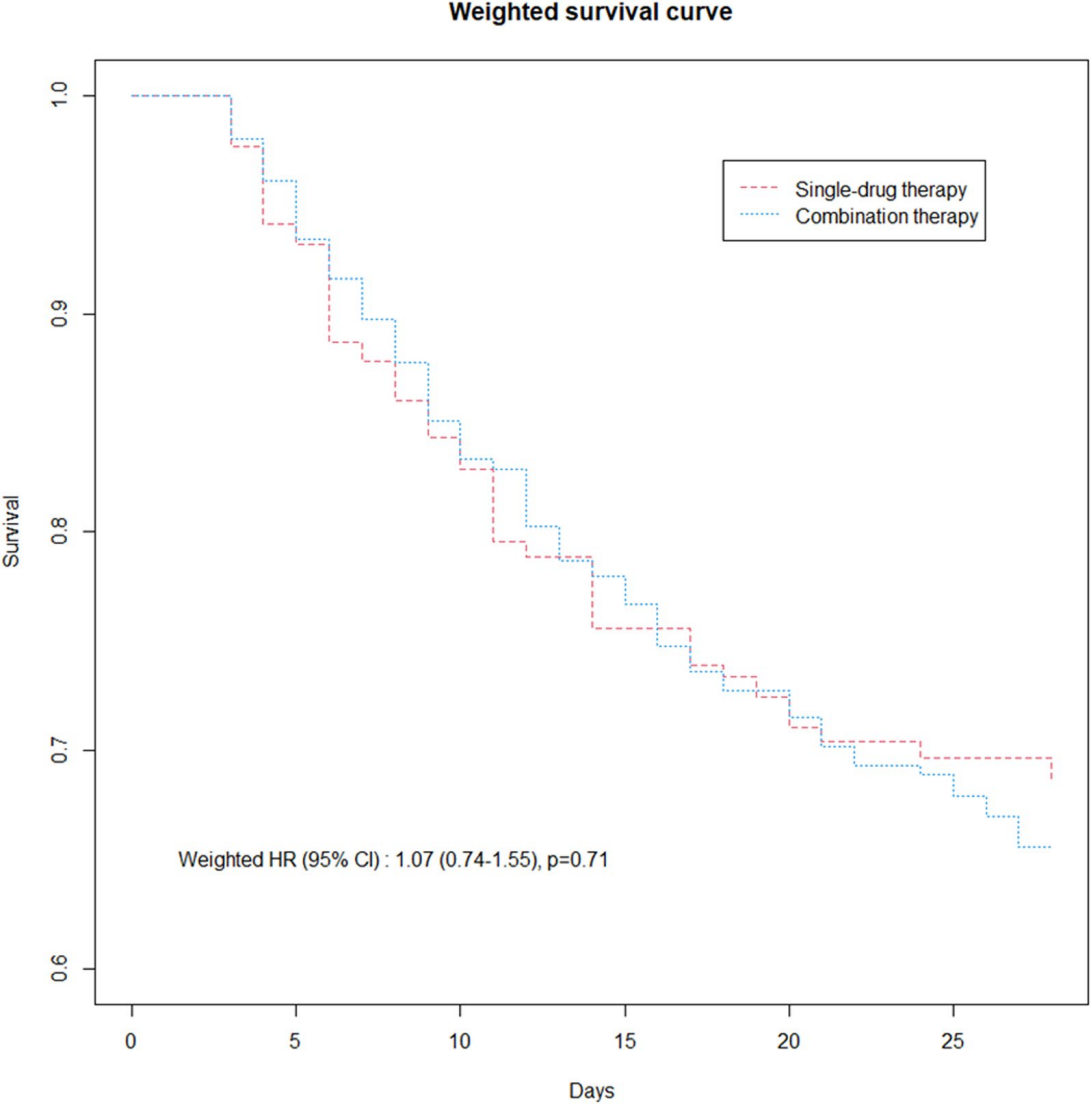
IPTW-adjusted cumulative survival at Day 28 in patients treated with single-drug versus combination therapy. IPTW, inverse probability of treatment weighting; HR, hazard ratio; CI, confidence interval

**Fig. 2:**
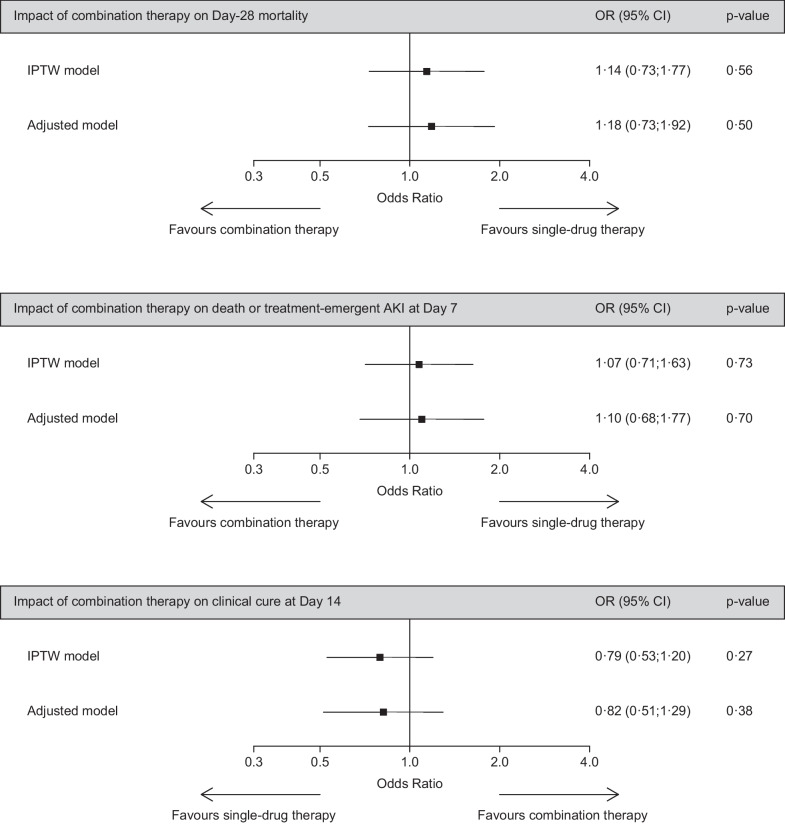
Impact of combination therapy on study endpoints: results of IPTW and multivariable regression analyses (whole study population). Regression models were adjusted on inclusion period, the SAPS 2 score value at intensive care unit admission, prior chronic diseases, prior immune deficiency, time from hospital admission to pneumonia, pneumonia type, and the SOFA score value at pneumonia onset. In addition, (i) the model focused on clinical cure at Day 14 was adjusted on colonization with MDR Gram-negative bacteria, and (ii) the model focused on death or treatment-emergent AKI at Day 7 was adjusted on diabetes mellitus, prior contrast-enhanced computerized tomography and/or angiography, and prior aminoglycoside and/or glycopeptide exposure. See Additional file 1: Table S1 and Figure S1. OR, odds ratio; CI, confidence interval; IPTW, inverse probability of treatment weighting; AKI, acute kidney injury

All-cause mortality rates at Day 28 were 28.5% and 32.9% in the single-drug and combination groups, respectively (*P* = 0.36) (Table 1). The cumulative survival overtime did not significantly differ between the two groups (IPT-weighted hazard ratio, 1.07; 95% CI, 0.74–1.55; *P* = 0.71) (Fig. 1). After adjustment on potential confounders (Additional file 1: Table S1), combination therapy exerted no independent effect on this outcome, in IPTW analysis (aOR, 1.14; 95% CI, 0.73–1.77; *P* = 0.56) as in multivariable regression (aOR, 1.18; 95% CI, 0.73–1.92; *P* = 0.50) (Fig. 2). This lack of association between combination therapy and Day-28 mortality was confirmed in subgroup analyses focused on patients with non-carbapenem-based regimen, aminoglycoside as companion drug, combination therapy administered for ≥ 3 days, pneumonia due to MDR and/or non-fermenting Gram-negative bacteria, and a SOFA score value ≥ 7 or septic shock at pneumonia onset (Table 2).

**Table 2:** Impact of combination therapy on study endpoints: results of subgroup analyses

Patient subpopulations	Mortality at Day 28		Clinical cure at Day 14		Death or AKI at Day 7	
	aOR (95% CI)	*P*-value	aOR (95% CI)	*P*-value	aOR (95% CI)	*P*-value
Pneumonia due to MDR Gram-negative bacteria	0.88 (0.31–2.53)	0.82	1.52 (0.42–5.41)	0.52	1.82 (0.57–5.77)	0.31
Pneumonia due to non-MDR Gram-negative bacteria	1.22 (0.69–2.16)	0.50	0.76 (0.46–1.27)	0.30	0.96 (0.55–1.66)	0.88
Pneumonia due to no-fermenting Gram-negative bacteria	0.73 (0.30–1.73)	0.47	1.13 (0.49–2.56)	0.78	1.36 (0.54–3.46)	0.52
Carbapenem-based regimen	1.14 (0.45–2.88)	0.78	0.53 (0.20–1.41)	0.21	1.51 (0.45–5.05)	0.50
Non-carbapenem-based regimen	1.09 (0.60–1.99)	0.77	0.99 (0.57–1.73)	0.98	0.81 (0.46–1.44)	0.48
Aminoglycoside-containing regimen	1.23 (0.74–2.06)	0.42	0.78 (0.48–1.26)	0.31	1.05 (0.62–1.76)	0.86
Non-aminoglycoside-containing regimen	1.01 (0.48–2.12)	0.98	0.76 (0.39–1.47)	0.42	1.26 (0.65–2.46)	0.49
Combination therapy < 3 days	1.04 (0.58–1.87)	0.90	1.12 (0.64–1.95)	0.70	1.00 (0.55–1.80)	0.99
Combination therapy ≥ 3 days	1.34 (0.76–2.39)	0.32	0.59 (0.35–1.01)	0.05	1.18 (0.68–2.05)	0.55
SOFA score value < 7 at pneumonia onset	1.43 (0.65–3.12)	0.37	0.79 (0.42–1.50)	0.47	1.11 (0.56–2.18)	0.77
SOFA score value ≥ 7 at pneumonia onset	1.01 (0.54–1.91)	0.97	0.89 (0.43–1.84)	0.76	1.02 (0.50–2.09)	0.95
Septic shock at pneumonia onset	1.40 (0.49–3.99)	0.53	0.60 (0.19–1.88)	0.38	2.22 (0.65–7.62)	0.21
Pneumonia-related BSI ^a^	1.49 (0.29–7.74)	0.64	0.50 (0.10–2.43)	0.39	0.80 (0.17–3.77)	0.78

Multivariate regression models were adjusted on inclusion period, the SAPS 2 score value at intensive care unit admission, prior chronic diseases, prior immune deficiency, time from hospital admission to pneumonia, pneumonia type, and the SOFA score value at pneumonia onset. In addition, (i) the model focused on clinical cure at Day 14 was adjusted on colonization with MDR Gram-negative bacteria, and (ii) the model focused on death or treatment-emergent AKI at Day 7 was adjusted on diabetes mellitus, prior contrast-enhanced computerized tomography and/or angiography, and prior aminoglycoside and/or glycopeptide exposure. See Additional file 1: Table S1 and Figure S1

AKI, acute kidney injury; aOR, adjusted odd ratio; CI, confidence interval; MDR, multidrug-resistant; SOFA, sepsis-related organ failure assessment; BSI, bloodstream infection

^a^Univariate analysis due to the low number of patients with pneumonia-related BSI

### Impact of combination therapy on clinical cure at day 14

There was a trend toward a higher clinical cure rate at Day 14 in patients receiving single-drug therapy when compared to those treated with combination therapy (49.7% versus 40.0%, *P* = 0.06) (Table 1). Combination therapy was not independently associated with this endpoint, in IPTW analysis (aOR, 0.79; 95% CI 0.53–1.20; *P* = 0.27) as in multivariable regression (aOR, 0.82; 95% CI 0.51–1.29; *P* = 0.38) (Fig. 1). In subgroup analyses, receiving combination therapy for ≥ 3 days was negatively linked with the likelihood of clinical cure at Day 14 (Table 2). No association was observed between combination therapy and this endpoint in all other subgroups, including in patients infected with MDR and/or non-fermenting Gram-negative bacteria (Table 2).

### Impact of combination therapy on the hazard of death or treatment-emergent AKI at day 7

The cumulative incidences of treatment-emergent AKI (39.6% versus 31.1%, *P* = 0.09) and death (10.0% versus 10.6%, *P* = 0.85) at Day 7 did not significantly differ between the combination and the single-drug group. In the combination group, the cumulative incidence of AKI was similar in patients receiving aminoglycosides and those receiving other companion drugs (66/174 = 37.9% versus 29/66 = 43.9%, *P* = 0.30) (Table 1). Combination therapy exerted no independent effect on the likelihood of AKI or death at Day 7, in IPTW analysis (aOR, 1.07; 95% CI 0.71–1.63; *P* = 0.73) as in multivariable regression (aOR, 1.10; 95% CI, 0.68–1.77; *P* = 0.70) (Fig. 1). Likewise, combination therapy was not associated with this composite outcome in analyses restricted to patients receiving an aminoglycoside as companion drug, those receiving combination for ≥ 3 days, and in those with a SOFA score value ≥ 7 points or septic shock at pneumonia onset (Table 2).

## Discussion

In this multicenter study including 391 critically ill patients with HAP, vHAP or VAP due to Gram-negative pathogens, the initial administration of adequate combination therapy was not independently associated with a reduced hazard of death at Day 28 or a greater likelihood of clinical cure at Day 14 when compared to adequate single-drug regimen. No over-risk of treatment-emergent AKI was observed in patients receiving combination therapy.

A meta-analysis of randomized controlled trials published fifteen years ago failed to demonstrate a benefit of initial combination therapy on short-term survival in patients with suspected VAP [7]. Yet, most of trials included a low proportion of patients with potentially difficult-to-treat pathogens (namely, non-fermenting and/or MDR Gram-negative bacteria) and used heterogeneous definitions for septic shock. Moreover, no subgroup analysis was performed in patients with microbiologically documented VAP and receiving adequate single-drug or combination therapy [7]. A more recent randomized trial showed no difference on Day-28 mortality in patients with suspected VAP treated with meropenem plus ciprofloxacin versus meropenem alone; however, a large subset of the study population had either no confirmed VAP or VAP involving pathogens without therapeutic issue (e.g., oropharyngeal flora) [8]. Also, the potential effect of combination therapy was not investigated according to baseline severity [8]. Another trial comparing meropenem plus moxifloxacin versus meropenem alone in patients with sepsis found no difference on survival or the course of organ failures, including in the subgroup with pneumonia; nevertheless, only one third of patients had a microbiologically confirmed infection at enrollment [28]. Overall, while the delayed administration of active antimicrobials has been repeatedly associated with impaired outcomes in critically ill patients with culture-proven HAP or VAP [29, 30], it remains unsettled whether adequate combination therapy may improve survival in this population when compared to adequate single-drug therapy. This may contribute to intensivists’ reluctance for de-escalation and cessation of the companion drug in this situation [31]. Evidence on this issue is almost inexistent for patients with vHAP, who consistently exhibit a worse prognosis than those with VAP or HAP [32–34]. In our study, receiving combination therapy was not associated with Day-28 mortality, in univariate analyses as after adjustment on potential confounders. These results indicate that dual-active regimen provide no survival gain in patients with nosocomial pneumonia due to Gram-negative pathogens, notably in those with vHAP. This is consistent with current guidelines advocating empirical combination therapy in severely ill patients with HAP or VAP and risk factors for MDR Gram-negative pathogens then de-escalation to a single-drug regimen once susceptibility test results become available [4, 5].

The prevalence of sepsis and septic shock at pneumonia onset was higher in the combination group, which may merely reflect that intensivists were more prone to prescribe initial—that is, most often empirical—combination therapy in patients with these conditions, in concordance with academic recommendations [4, 5]. However, whether the most severely ill patients may still benefit from combination therapy is an unsolved question [1, 2]. A meta-analysis of randomized trials including only patients with sepsis—related or not to pulmonary infections—found no survival improvement with combination therapy; however, the quantity and quality of data was low, precluding any definite conclusion to be drawn [3]. Besides, certain observational studies reported improved outcomes with combination therapy in patients at high baseline risk for death [15, 16, 35]. The subgroup analyses performed as part of our work suggest that combination therapy exerts no effect on survival in patients with septic shock, severe hypoxemia or, more globally, high SOFA score values at pneumonia onset provided that the pivotal β-lactam is fully active against the causative Gram-negative pathogen. A major aim of empirical combination therapy is to increase the likelihood of administering at least one agent with in vitro activity against the causative pathogen [4, 5, 10, 36]. Hence, our data do not support the use of empirical combination therapy in patients without risk factors for pneumonia due to MDR pathogens, including in those with the most severe presentations. Adequately powered prospective trials are warranted to confirm these results and reinforce antimicrobial stewardship guidelines in such situations.

This lack of survival impact could plausibly result from a counterbalance between a benefit in terms of clinical response and an increased incidence of severe antimicrobial-related adverse events—especially AKI when nephrotoxic agents are administered as companion drugs [13, 14, 37]. Our data argue against this hypothesis. First, and in concordance with the available evidence [7, 10, 12, 38], combination therapy was not associated with a greater likelihood of clinical cure, including in patients infected with non-fermenting Gram-negative pathogens or in those receiving second agents with substantial lung bioavailability (*i.e*., fluoroquinolones or cotrimoxazole). Conversely, there was a trend toward a higher clinical cure rate in the single-drug group; moreover, receiving combination therapy for 3 days or more was negatively linked with the likelihood of clinical cure in adjusted analyses. Yet, these findings may merely reflect intensivists’ decision to continue the companion drug in patients with unfavorable clinical response at Day 3. Next, the crude incidence of treatment-emergent AKI was slightly higher in the combination group, which was likely due to a more common use of dual regimen in the most severe patients—i.e. those with the more pronounced hazard of renal failure, independently of drug exposure. However, combination therapy with an aminoglycoside was not associated with this outcome after careful adjustment on potential confounders, including severity indexes and other predisposing factors for AKI. This result may ensue from the routine use of once-daily administration, monitoring of trough concentrations, and other measures to prevent aminoglycoside-induced nephrotoxicity in the participating ICUs [39–41].

This work has limitations. First, this is an observational study. Therefore, despite the use of prospectively collected data and PS-adjusted analyses, residual confounding on the outcomes of interest cannot be firmly ruled out. Second, the potential impact of dosing schemes on the study endpoints was not investigated due to the limited subset of patients with each given regimen. Also, information on therapeutic drug monitoring was not available in the database. However, initial daily doses of pivotal and companion drugs were consistent with current standards and guidelines for optimized antimicrobial pharmacokinetic in critically ill individuals (Additional file 1: Table S2) [2, 4, 5]. Along this line, single-drug therapies with an aminoglycoside or colistin were considered as adequate when fully active in vitro though the pulmonary diffusion of these agents may be suboptimal with low-dose regimen. Fourth, the outcome effect of combination therapy in pneumonia involving XDR or PDR Gram-negative bacteria could not be appraised due to the very low number of patients infected with such pathogens—dedicated studies are warranted to solve this question, especially with novel β-lactams [35, 42, 43]. Fifth, we compared the hazard of AKI between patients with combination and single-drug therapy; yet, adding a second agent to an effective pivotal drug may cause other antimicrobial-related side-effects that were not investigated in our work [44, 45]. Sixth, we only studied the first pneumonia in patients with multiple episodes during the ICU stay. Whether patients with pneumonia relapse or second VAP may benefit from combination therapy remains to be explored. Lastly, we did not appraise the impact of combination therapy in patients with HAP managed outside the ICU.

## Conclusion

When compared to initial adequate single-drug regimen, initial adequate combination therapy exerts no independent impact on survival or clinical success rate in critically ill patients with a first episode of mono-bacterial HAP, vHAP or VAP due to Gram-negative pathogens, including in those with pneumonia due to non-fermenting bacteria or presenting with septic shock, severe hypoxemia or high SOFA score values at infection onset.

### Supplementary Information

**Additional file 1. **Supplementary tables and figures.

## Data Availability

The data that support the findings of this study are available on reasonable request to the corresponding author.

## Abbreviations

AIDS: Acquired immune deficiency syndrome
AKI: Acute kidney injury
aOR: Adjusted odd ratio
BMI: Body mass index
CFU: Colony-forming unit
CI: Confidence interval
CT: Computerized tomography
ECMO: Renal replacement therapy
HAP: Hospital-acquired pneumonia
ICU: Intensive care unit
IPTW: Inverse probability of treatment weighting
LOS: Length of stay
MDRB: Multidrug-resistant bacteria
MV: Mechanical ventilation
PaO_2_/FiO_2_: Arterial oxygen partial pressure/inspired fraction of oxygen
PDR: Pan-drug-resistant
PS: Propensity score
RRT: Renal replacement therapy
SAPS 2: Simplified acute physiology score 2
SOFA: Sepsis-related organ failure assessment
STROBE: Strengthening the Reporting of Observational Studies in Epidemiology
VAP: Ventilator-associated pneumonia
vHAP: Ventilated HAP
XDR: Extensively drug-resistant

## References

[1] Coopersmith CM, De Backer D, Deutschman CS, Ferrer R, Lat I, Machado FR (2018). Surviving Sepsis Campaign: research priorities for sepsis and septic shock. Crit Care Med.

[2] Timsit JF, Bassetti M, Cremer O, Daikos G, de Waele J, Kallil A (2019). Rationalizing antimicrobial therapy in the ICU: a narrative review. Intensive Care Med.

[3] Sjövall F, Perner A, Hylander MM (2017). Empirical mono- versus combination antibiotic therapy in adult intensive care patients with severe sepsis—a systematic review with meta-analysis and trial sequential analysis. J Infect.

[4] Torres A, Niederman MS, Chastre J, Ewig S, Fernandez-Vandellos P, Hanberger H (2017). International ERS/ESICM/ESCMID/ALAT guidelines for the management of hospital-acquired pneumonia and ventilator-associated pneumonia: Guidelines for the management of hospital-acquired pneumonia (HAP)/ventilator-associated pneumonia (VAP) of the European Respiratory Society (ERS), European Society of Intensive Care Medicine (ESICM), European Society of Clinical Microbiology and Infectious Diseases (ESCMID) and Asociación Latinoamericana del Tórax (ALAT). Eur Respir J.

[5] Kalil AC, Metersky ML, Klompas M, Muscedere J, Sweeney DA, Palmer LB (2016). Management of adults with hospital-acquired and ventilator-associated pneumonia: 2016 clinical practice guidelines by the Infectious Diseases Society of America and the American Thoracic Society. Clin Infect Dis.

[6] Tamma PD, Cosgrove SE, Maragakis LL (2012). Combination therapy for treatment of infections with Gram-negative bacteria. Clin Microbiol Rev.

[7] Aarts MAW, Hancock JN, Heyland D, McLeod RS, Marshall JC (2008). Empiric antibiotic therapy for suspected ventilator-associated pneumonia: a systematic review and meta-analysis of randomized trials. Crit Care Med.

[8] Heyland DK, Dodek P, Muscedere J, Day A, Cook D, Canadian Critical Care Trials Group (2008). Randomized trial of combination versus monotherapy for the empiric treatment of suspected ventilator-associated pneumonia. Crit Care Med.

[9] Bliziotis IA, Samonis G, Vardakas KZ, Chrysanthopoulou S, Falagas ME (2005). Effect of aminoglycoside and beta-lactam combination therapy versus beta-lactam monotherapy on the emergence of antimicrobial resistance: a meta-analysis of randomized, controlled trials. Clin Infect Dis.

[10] Garnacho-Montero J, Sa-Borges M, Sole-Violan J, Barcenilla F, Escoresca-Ortega A, Ochoa M (2007). Optimal management therapy for *Pseudomonas aeruginosa* ventilator-associated pneumonia: an observational, multicenter study comparing monotherapy with combination antibiotic therapy. Crit Care Med.

[11] Deconinck L, Meybeck A, Patoz P, Van Grunderbeeck N, Boussekey N, Chiche A (2017). Impact of combination therapy and early de-escalation on outcome of ventilator-associated pneumonia caused by *Pseudomonas aeruginosa*. Infect Dis Lond Engl.

[12] Foucrier A, Dessalle T, Tuffet S, Federici L, Dahyot-Fizelier C, Barbier F (2023). Association between combination antibiotic therapy as opposed as monotherapy and outcomes of ICU patients with *Pseudomonas aeruginosa* ventilator-associated pneumonia: an ancillary study of the iDIAPASON trial. Crit Care.

[13] Ong DSY, Frencken JF, Klein Klouwenberg PMC, Juffermans N, van der Poll T, Bonten MJM (2017). Short-course adjunctive gentamicin as empirical therapy in patients with severe sepsis and septic shock: a prospective observational cohort study. Clin Infect Dis.

[14] Paul M, Lador A, Grozinsky-Glasberg S, Leibovici L (2014). Beta lactam antibiotic monotherapy versus beta lactam-aminoglycoside antibiotic combination therapy for sepsis. Cochrane Database Syst Rev.

[15] Kumar A, Safdar N, Kethireddy S, Chateau D (2010). A survival benefit of combination antibiotic therapy for serious infections associated with sepsis and septic shock is contingent only on the risk of death: a meta-analytic/meta-regression study. Crit Care Med.

[16] Ripa M, Rodríguez-Núñez O, Cardozo C, Naharro-Abellán A, Almela M, Marco F (2017). Influence of empirical double-active combination antimicrobial therapy compared with active monotherapy on mortality in patients with septic shock: a propensity score-adjusted and matched analysis. J Antimicrob Chemother.

[17] Park SY, Park HJ, Moon SM, Park KH, Chong YP, Kim MN (2012). Impact of adequate empirical combination therapy on mortality from bacteremic Pseudomonas aeruginosa pneumonia. BMC Infect Dis.

[18] Barbier F, Pommier C, Essaied W, Garrouste-Orgeas M, Schwebel C, Ruckly S (2016). Colonization and infection with extended-spectrum β-lactamase-producing Enterobacteriaceae in ICU patients: what impact on outcomes and carbapenem exposure?. J Antimicrob Chemother.

[19] von Elm E, Altman DG, Egger M, Pocock SJ, Gøtzsche PC, Vandenbroucke JP (2007). The strengthening the reporting of observational studies in epidemiology (STROBE) statement: guidelines for reporting observational studies. Lancet.

[20] Vallecoccia MS, Dominedò C, Cutuli SL, Martin-Loeches I, Torres A, De Pascale G (2020). Is ventilated hospital-acquired pneumonia a worse entity than ventilator-associated pneumonia?. Eur Respir Rev.

[21] Tabah A, Bassetti M, Kollef MH, Zahar JR, Paiva JA, Timsit JF (2020). Antimicrobial de-escalation in critically ill patients: a position statement from a task force of the European Society of Intensive Care Medicine (ESICM) and European Society of Clinical Microbiology and Infectious Diseases (ESCMID) Critically Ill Patients Study Group (ESGCIP). Intensive Care Med.

[22] Magiorakos AP, Srinivasan A, Carey RB, Carmeli Y, Falagas ME, Giske CG (2012). Multidrug-resistant, extensively drug-resistant and pandrug-resistant bacteria: an international expert proposal for interim standard definitions for acquired resistance. Clin Microbiol Infect.

[23] Singer M, Deutschman CS, Seymour CW, Shankar-Hari M, Annane D, Bauer M (2016). The third international consensus definitions for sepsis and septic shock (Sepsis-3). JAMA.

[24] Weiss E, Zahar JR, Alder J, Asehnoune K, Bassetti M, Bonten MJM (2019). Elaboration of consensus clinical endpoints to evaluate antimicrobial treatment efficacy in future hospital-acquired/ventilator-associated bacterial pneumonia clinical trials. Clin Infect Dis.

[25] Khwaja A (2012). KDIGO clinical practice guidelines for acute kidney injury. Nephron Clin Pract.

[26] See CY, Pan HC, Chen JY, Wu CY, Liao HW, Huang YT (2023). Improvement of composite kidney outcomes by AKI care bundles: a systematic review and meta-analysis. Crit Care Lond Engl.

[27] Austin PC, Stuart EA (2015). Moving towards best practice when using inverse probability of treatment weighting (IPTW) using the propensity score to estimate causal treatment effects in observational studies. Stat Med.

[28] Brunkhorst FM, Oppert M, Marx G, Bloos F, Ludewig K, Putensen C (2012). Effect of empirical treatment with moxifloxacin and meropenem vs meropenem on sepsis-related organ dysfunction in patients with severe sepsis: a randomized trial. JAMA.

[29] Kuti EL, Patel AA, Coleman CI (2008). Impact of inappropriate antibiotic therapy on mortality in patients with ventilator-associated pneumonia and blood stream infection: a meta-analysis. J Crit Care.

[30] Jovanovic B, Djuric O, Hadzibegovic A, Jovanovic S, Stanisavljevic J, Milenkovic M (2021). Trauma and antimicrobial resistance are independent predictors of inadequate empirical antimicrobial treatment of ventilator-associated pneumonia in critically ill patients. Surg Infect.

[31] Roquilly A, Chanques G, Lasocki S, Foucrier A, Fermier B, De Courson H (2021). Implementation of French recommendations for the prevention and the treatment of hospital-acquired pneumonia: a cluster-randomized trial. Clin Infect Dis.

[32] Ibn Saied W, Mourvillier B, Cohen Y, Ruckly S, Reignier J, Marcotte G (2019). A comparison of the mortality risk associated with ventilator-acquired bacterial pneumonia and nonventilator ICU-acquired bacterial pneumonia. Crit Care Med.

[33] Talbot GH, Das A, Cush S, Dane A, Wible M, Echols R (2019). Evidence-based study design for hospital-acquired bacterial pneumonia and ventilator-associated bacterial pneumonia. J Infect Dis.

[34] Esperatti M, Ferrer M, Theessen A, Liapikou A, Valencia M, Saucedo LM (2010). Nosocomial pneumonia in the intensive care unit acquired by mechanically ventilated versus nonventilated patients. Am J Respir Crit Care Med.

[35] Gutiérrez-Gutiérrez B, Salamanca E, de Cueto M, Hsueh PR, Viale P, Paño-Pardo JR (2017). Effect of appropriate combination therapy on mortality of patients with bloodstream infections due to carbapenemase-producing Enterobacteriaceae (INCREMENT): a retrospective cohort study. Lancet Infect Dis.

[36] Micek ST, Reichley RM, Kollef MH (2011). Health care-associated pneumonia (HCAP): empiric antibiotics targeting methicillin-resistant *Staphylococcus aureus* (MRSA) and *Pseudomonas aeruginosa* predict optimal outcome. Medicine (Baltimore).

[37] Kett DH, Cano E, Quartin AA, Mangino JE, Zervos MJ, Peyrani P (2011). Implementation of guidelines for management of possible multidrug-resistant pneumonia in intensive care: an observational, multicentre cohort study. Lancet Infect Dis.

[38] Planquette B, Timsit JF, Misset BY, Schwebel C, Azoulay E, Adrie C (2013). Pseudomonas aeruginosa ventilator-associated pneumonia predictive factors of treatment failure. Am J Respir Crit Care Med.

[39] Bartal C, Danon A, Schlaeffer F, Reisenberg K, Alkan M, Smoliakov R (2003). Pharmacokinetic dosing of aminoglycosides: a controlled trial. Am J Med.

[40] Gálvez R, Luengo C, Cornejo R, Kosche J, Romero C, Tobar E (2011). Higher than recommended amikacin loading doses achieve pharmacokinetic targets without associated toxicity. Int J Antimicrob Agents.

[41] The International Antimicrobial Therapy Cooperative Group of the European Organization for Research and Treatment of Cancer. Efficacy and toxicity of single daily doses of amikacin and ceftriaxone versus multiple daily doses of amikacin and ceftazidime for infection in patients with cancer and granulocytopenia. Ann Intern Med. 1993;119(7 Pt 1):584–93. 10.7326/0003-4819-119-7_Part_1-199310010-00006 8363169

[42] Durante-Mangoni E, Andini R, Zampino R (2019). Management of carbapenem-resistant Enterobacteriaceae infections. Clin Microbiol Infect.

[43] Barbier F, Hraiech S, Kernéis S, Veluppillai N, Pajot O, Poissy J (2023). Rationale and evidence for the use of new beta-lactam/beta-lactamase inhibitor combinations and cefiderocol in critically ill patients. Ann Intensive Care.

[44] Arulkumaran N, Routledge M, Schlebusch S, Lipman J, Conway MA (2020). Antimicrobial-associated harm in critical care: a narrative review. Intensive Care Med.

[45] Rhee C, Kadri SS, Dekker JP, Danner RL, Chen HC, Fram D (2020). Prevalence of antibiotic-resistant pathogens in culture-proven sepsis and outcomes associated with inadequate and broad-spectrum empiric antibiotic use. JAMA Netw Open.

