# Supplementary material for: Single-drug versus combination antimicrobial therapy in critically ill patients with hospital-acquired pneumonia and ventilator-associated pneumonia due to Gram-negative pathogens: a multicenter retrospective cohort study
Source: Crit Care. 2024 Jan 3;28:10. doi: 10.1186/s13054-023-04792-0 (PMC10765858; doi:10.1186/s13054-023-04792-0)
Supplement: Supplementary file 1 — Additional file 1. Supplementary tables and figures. [file 13054_2023_4792_MOESM1_ESM.docx]

**ELECTRONIC SUPPLEMENTARY MATERIAL**

**Single-drug versus combination antimicrobial therapy in critically ill patients with hospital-acquired pneumonia and ventilator-associated pneumonia due to Gram-negative pathogens: a multicenter retrospective cohort study**

François Barbier, MD PhD; Claire Dupuis, MD PhD; Niccolò Buetti, MD PhD; Carole Schwebel, MD PhD; Élie Azoulay, MD PhD; Laurent Argaud, MD PhD; Yves Cohen, MD PhD; Vivien Hong Tuan Ha, MD; Marc Gainnier, MD PhD; Shidasp Siami, MD PhD; Jean-Marie Forel, MD PhD; Christophe Adrie, MD PhD; Étienne de Montmollin, MD PhD; Jean Reignier, MD PhD; Stéphane Ruckly, MSc; Jean-Ralph Zahar, MD PhD; Jean-François Timsit, MD PhD; on behalf of the OutcomeRéa Study Group

**Table S1.** Propensity score-based calculation of inverse probability of combination therapy weighting according to study endpoints

| **Variables** | **df** | **Estimate** | **OR (95% CI)** | ***P*-value** |
| --- | --- | --- | --- | --- |
| **Endpoints: death at Day 28** | | | | |
| **Admission period** |  |  |  |  |
| 2008-2011 |  |  | 1 | 0.0005 |
| 2012-2015 | 1 | -0.1329 | 0.88 (0.55 - 1.39) |  |
| 2016-2019 | 1 | -1.2661 | 0.28 (0.15 – 0.54) |  |
| **Chronic diseases** |  |  |  |  |
| Any, except immune deficiency | 1 | 0.1472 | 1.16 (0.75 – 1.79) | 0.5094 |
| Immune deficiency | 1 | 0.0497 | 1.05 (0.63 – 1.76) | 0.8500 |
| **SAPS 2 at ICU admission** | 1 | -0.0113 | 0.99 (0.98 - 1.00) | 0.0550 |
| **Pneumonia classification** |  |  |  |  |
| vHAP |  |  | 1 | 0.0980 |
| HAP | 1 | -0.8660 | 0.42 (0.19 – 0.94) |  |
| VAP | 1 | -0.5472 | 0.58 (0.31 – 1.09) |  |
| **Characteristics of pneumonia** |  |  |  |  |
| Time from hospital admission >7 days | 1 | 0.4685 | 1.60 (1.00 – 2.54) | 0.0484 |
| SOFA score value at pneumonia onset | 1 | 0.0416 | 1.04 (0.98 – 1.11) | 0.1646 |
| Area under the receiver operating curve = 0.66  Hosmer-Lemeshow test, *P* = 0.86 | | | | |
| **Endpoint: clinical success at Day 14** |  |  |  |  |
| **Admission period** |  |  |  |  |
| 2008-2011 |  |  | 1 | 0.0004 |
| 2012-2015 | 1 | -0.1399 | 0.87 (0.55 – 1.39) |  |
| 2016-2019 | 1 | -1.3089 | 0.27 (0.14 – 0.52) |  |
| **Chronic diseases** |  |  |  |  |
| Any, except immune deficiency | 1 | 0.1118 | 1.12 (0.72 – 1.74) | 0.6191 |
| Immune deficiency | 1 | 0.0432 | 1.04 (0.62 – 1.75) | 0.8702 |
| **SAPS 2 at ICU admission** | 1 | -0.0119 | 0.99 (0.98 – 1.00) | 0.0451 |
| **Pneumonia classification** |  |  |  |  |
| vHAP |  |  | 1 | 0.0597 |
| HAP | 1 | -0.9517 | 0.39 (0.17 – 0.87) |  |
| VAP | 1 | -0.6406 | 0.53 (0.28 – 1.00) |  |
| **Characteristics of pneumonia** |  |  |  |  |
| Time from hospital admission >7 days | 1 | 0.4273 | 1.53 (0.96 – 2.45) | 0.0736 |
| SOFA score value at pneumonia onset | 1 | 0.0395 | 1.04 (0.98 – 1.10) | 0.1900 |
| **Pneumonia due to MDR GNB** | 1 | 0.7006 | 2.05 (0.95 – 4.28) | 0.0679 |
| Area under the receiver operating curve = 0.67  Hosmer-Lemeshow test, *P* = 0.81 | | | | |
| **Endpoint: death or acute kidney injury at Day 7** | | | | |
| **Admission period** |  |  |  |  |
| 2008-2011 |  |  | 1 | 0.0005 |
| 2012-2015 | 1 | -0.1303 | 0.88 (0.55 – 1.41) |  |
| 2016-2019 | 1 | -1.2701 | 0.28 (0.15 – 0.54) |  |
| **Chronic diseases** |  |  |  |  |
| Any, except immune deficiency | 1 | 0.1213 | 1.13 (0.72 – 1.76) | 0.5928 |
| Immune deficiency | 1 | 0.0643 | 1.07 (0.63 – 1.81) | 0.8118 |
| **SAPS 2 at ICU admission** | 1 | -0.0111 | 0.99 (0.98 – 1.00) | 0.0613 |
| **Pneumonia classification** |  |  |  |  |
| vHAP |  |  | 1 | 0.1385 |
| HAP | 1 | -0.8243 | 0.44 (0.19 – 0.99) |  |
| VAP | 1 | -0.4821 | 0.62 (0.23 – 1.21) |  |
| **Characteristics of pneumonia** |  |  |  |  |
| Time from hospital admission >7 days | 1 | 0.4997 | 1.65 (1.02 – 2.66) | 0.0409 |
| SOFA score value at pneumonia onset | 1 | 0.0435 | 1.04 (0.98 – 1.11) | 0.1562 |
| **Diabetes mellitus** | 1 | 0.1088 | 1.12 (0.64 – 1.94) | 0.6990 |
| **CECT and/or angiography** ^a^ | 1 | -0.1391 | 0.87 (0.54 – 1.41) | 0.5724 |
| **Glycopeptide exposure** ^a^ | 1 | -0.0529 | 0.95 (0.35 – 2.57) | 0.9172 |
| **Aminoglycoside exposure** ^a^ | 1 | -0.0361 | 0.97 (0.50 – 1.85) | 0.9138 |
| Area under the receiver operating curve = 0.66  Hosmer-Lemeshow test, *P* = 0.38 | | | | |

*Table S1 footnote*

df, degree of freedom; aOR, adjusted odd ratio; CI, confidence interval; AKI, acute kidney injury; SAPS 2, simplified acute physiology score 2; ICU, intensive care unit; vHAP, ventilated hospital-acquired pneumonia; HAP, non-ventilated hospital-acquired pneumonia; VAP, ventilator-associated pneumonia; SOFA, sepsis-related organ failure assessment; MDR GNB, multidrug-resistant Gram-negative bacteria; CECT, contrast-enhanced computerized tomography

^a^ Between ICU admission and pneumonia diagnosis (*i.e*., Day 0)

**Table S2.** Dosing schemes of the 10 most prescribed antimicrobial agents in the study cohort, according to renal function

| **Antimicrobial agents** |  | **Patients with available data (n) and**  **maximal daily doses at Day 0 / Day 1 (mg.kg^-1^ per 24 hours)** | | | | |  |
| --- | --- | --- | --- | --- | --- | --- | --- |
|  |  | **All patients** | **KDIGO 0** | **KDIGO 1** | **KDIGO 2** | **KDIGO 3** |  |
| Amoxicillin-clavulanate |  | 39 | 19 | 6 | 5 | 9 |  |
|  |  | 36.7  (25.6-55) | 46.9  (35.3-77.5) | 29.0  (25.6-42.9) | 32.6  (13-45.2) | 27.1  (9.8-40.5) |  |
| Cefotaxime |  | 16 | 6 | 1 | 3 | 6 |  |
|  |  | 54.3  (31.2-75.8) | 47.1  (38.5-70.6) | 94.2 | 46.4  (22.1-59.7) | 57.8  (22.3-81.1) |  |
| Ceftriaxone |  | 29 | 18 | 4 | 2 | 5 |  |
|  |  | 17.5  (13.6-24.1) | 22.7  (16.2-28.1) | 12.9  (11.4-18.5) | 12.0 & 13.7 | 17.5  (15.7-24.1) |  |
| Piperacillin-tazobactam |  | 83 | 36 | 11 | 14 | 22 |  |
|  |  | 157.9  (130.4-205.1) | 178.4  (145.6-225.5) | 170.2  (146.7-214.5) | 172.7  (154.8-200.0) | 133.3  (95.9-162.2) |  |
| Ceftazidime |  | 34 | 22 | 4 | 0 | 8 |  |
|  |  | 68.1  (53.7-85.1) | 63.5  (53.7-94.1) | 58.5  (36.7-87.2) | - | 71.8  (60.5-82.4) |  |
| Cefepime |  | 27 | 11 | 2 | 4 | 10 |  |
|  |  | 62.7  (44.4-80.4) | 70.6  (44.4-85.1) | 46.5 & 48.5 | 72.1  (59.0-83.7) | 52  (40.3-78.9) |  |
| Imipenem |  | 50 | 22 | 12 | 5 | 11 |  |
|  |  | 32.2  (22.0-46.2) | 35.0  (28.0-46.2) | 37.2  (20.3-43.5) | 34.1  (25.0-45.9) | 19.6  (12.1-31.9) |  |
| Meropenem |  | 15 | 5 | 2 | 1 | 7 |  |
|  |  | 41.7  (29.1-60.3) | 45.2  (41.7-71.9) | 56.1 & 60.3 | 23.7 | 40.2  (11.3-44.7) |  |
| Amikacin |  | 102 | 51 | 16 | 12 | 23 |  |
|  |  | 22.9  (19.4-25.8) | 21.3  (19.3-25.6) | 23.7  (17.8-28.1) | 20.7  (15.0-25.7) | 25.1  (22.5-28.2) |  |
| Ciprofloxacin |  | 26 | 15 | 4 | 1 | 6 |  |
|  |  | 12.5  (8.9-15.7) | 12.3  (7.9-15.7) | 14.1  (11.4-17.7) | 5.2 | 11.5  (8.9-15.7) |  |

*Table S2 footnote*

Daily antimicrobial doses are indicated as median (interquartile range), except for situations with <3 patients (in such situations, only individual values are mentioned).

Doses are exposed according to renal function, classified using the KDIGO (Kidney Disease: Improving Global Outcomes) nomenclature for acute kidney injury (KDIGO stage 0 indicates no renal failure) – see https://kdigo.org

**Figure S1.** Balance assessment of the models: unweighted and IPT-weighted absolute standardized differences

**Panel A:** death at Day 28


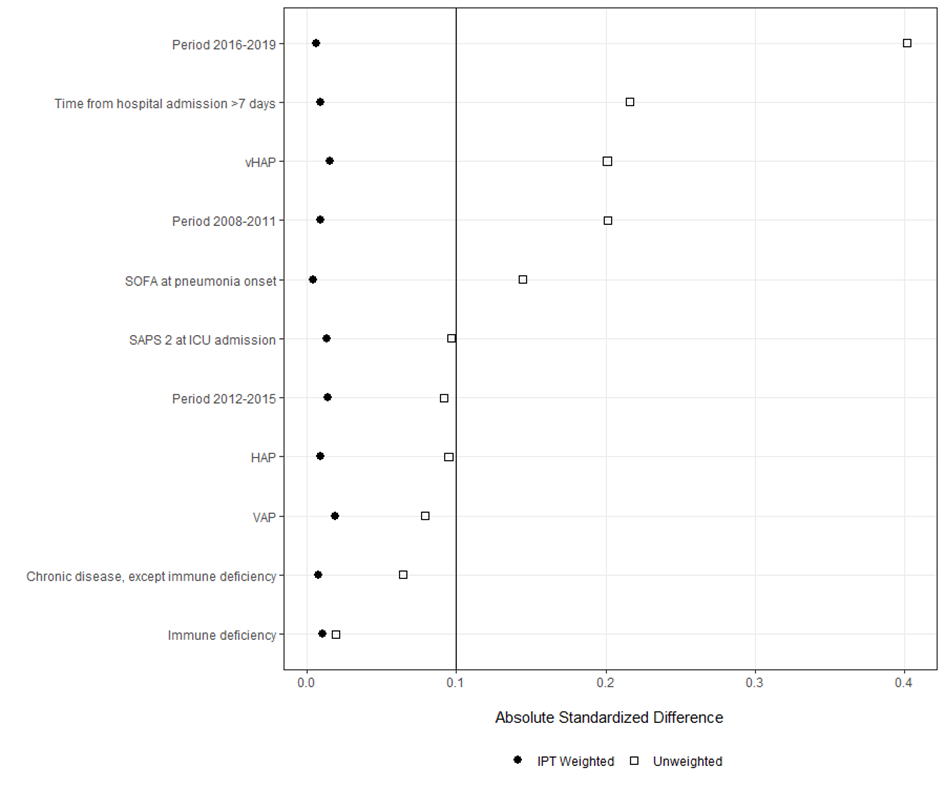


**Panel B:** clinical cure at Day 14


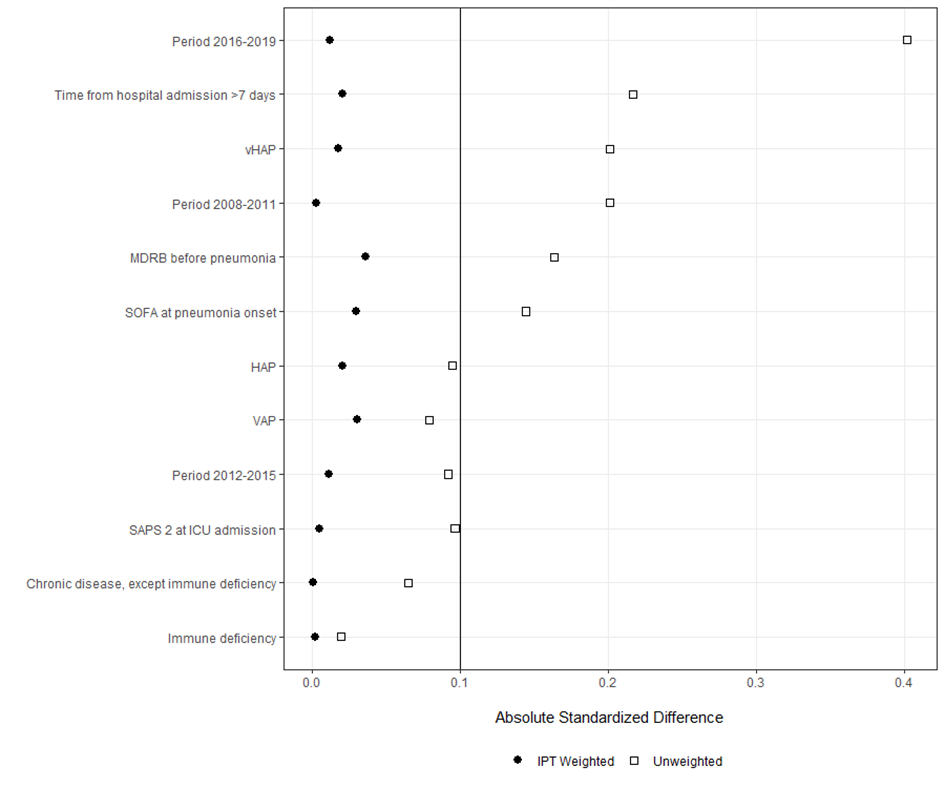


**Panel C:** death or acute kidney injury at Day 7

**
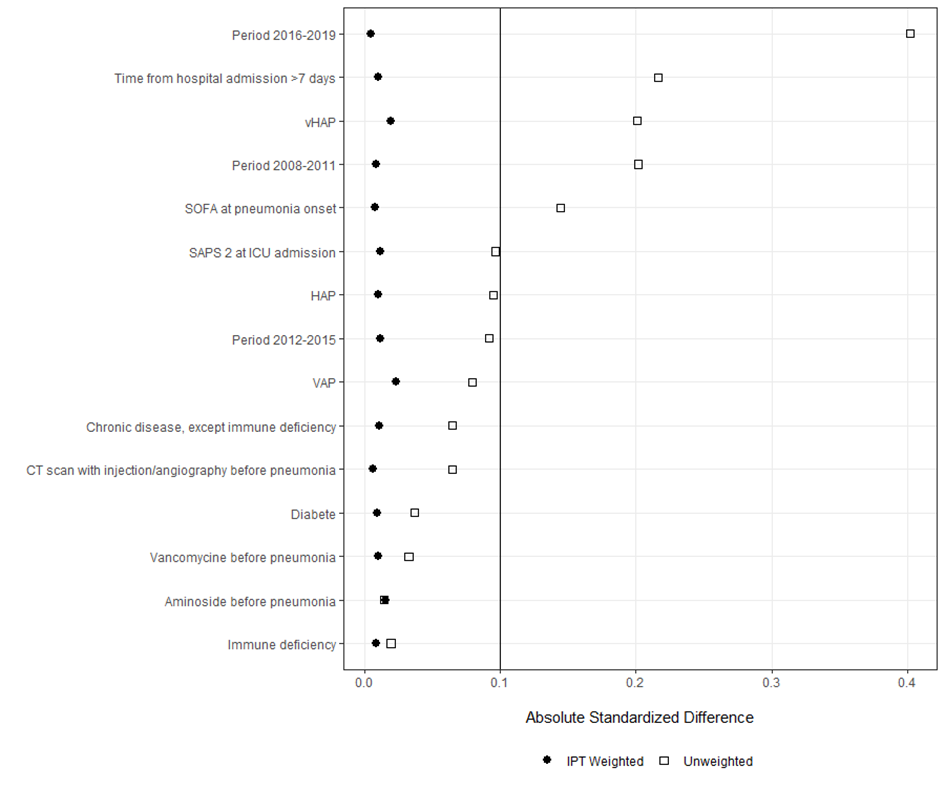
**

**Figure S2.** Study flowchart


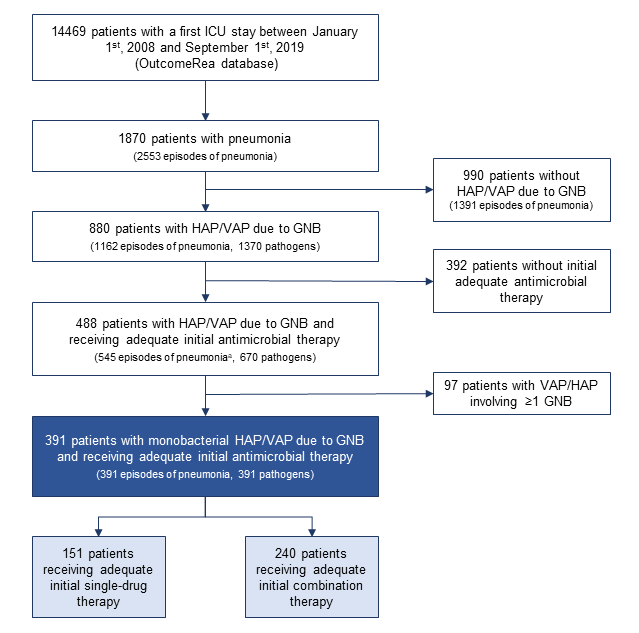


*Figure S2 footnote*

ICU, intensive care unit; HAP, hospital-acquired pneumonia; VAP, ventilator-associated pneumonia; GNB, Gram-negative bacteria

^a^ 57 patients had a second episode of pneumonia involving GNB and treated with initial adequate antimicrobial therapy during the ICU stay (in these patients, only the first episode was analyzed – see the Methods section for details).
